# ‘Civilising’ Deaf people in Tibet and Inner Mongolia: governing linguistic, ethnic and bodily difference in China

**DOI:** 10.1080/09687599.2017.1302319

**Published:** 2017-04-02

**Authors:** Theresia Hofer, Gry Sagli

**Affiliations:** ^a^ Department of Archaeology and Anthropology, University of Bristol, Bristol, UK; ^b^ Institute of Social and Cultural Anthropology, University of Oxford, Oxford, UK; ^c^ Department of International Studies and Interpreting, Oslo and Akershus University College of Applied Sciences, Oslo, Norway

**Keywords:** Ethnic sign languages, ethnicity, Tibet, Inner Mongolia, China, Deaf Studies

## Abstract

The People’s Republic of China is home to over 20 million d/Deaf and hard-of-hearing people, many among them belonging to ethnic minorities. Drawing on ethnographic fieldwork in two minority regions, the Tibet Autonomous Region and the Inner Mongolian Autonomous Region, this article comparatively discusses findings on sign language use, education and state welfare policies. The situation in these domains is analysed through the framework of the ‘civilising project’, coined by Harrell, and its impacts on the d/Deaf and hard-of-hearing among ethnic minorities are shown. For instance, through the promotion of Chinese and Chinese Sign Language over and above the use of local sign and written languages as well as through education and the medicalisation of disabilities.

## Points of interest

• There are 20 million deaf and hard-of-hearing people in China.
• Among them, many belong to ethnic minorities. This article focuses on deaf Tibetans and Mongolians.
• The role and status of minority sign languages in Tibet and Inner Mongolia is discussed.
• Tibetan Sign Language has achieved some official recognition by the Chinese state. The roles of local sign languages in Tibet and Inner Mongolia are overall very limited.
• Deaf Tibetans and deaf Mongolians in China are expected to learn to speak and write Chinese and not Tibetan and Mongolian languages.
• Deaf children in Tibet and Inner Mongolia are not granted the same right to ethnic minority languages as most hearing children are in the same regions.
• Special schools and rehabilitation centres teach deaf Tibetan and Mongolian children to read and speak Chinese.

## Introduction

Deaf Studies has so far mainly discussed the history, anthropology and politics of deafness and d/Deaf people in Europe, the United States and other places in the Global North.^1^ Only recently have researchers begun to offer in-depth ethnographies of and with deaf people worldwide, including the Global South (for example, Friedner 2015; Hoffmann-Dilloway 2010, 2016; Kusters 2014; Nakamura 2006; Nonaka 2014). No comparable work has been carried out in the People’s Republic of China (PRC),^2^ home to an estimated 20 million deaf and hard-of-hearing people (CDPF – China Disabled Persons’ Federation 2008). Whatever has been published relating to the deaf in China is typically about formal government education and grounded in research of areas populated by Han Chinese, who are the majority ethnic group in the country (Callaway 2000; Johnson 2003; Lytle, Johnson, and Hui [2005] 2006; Yang 2011). No studies have examined, or even mention, the situation of deaf people among non-Han groups. These are peoples who the government has classified as ‘ethnic minorities’ (*shaoshu minzu*) and who as such play a crucial role in the national imaginary and political unity of the PRC (Harrell 1995). Drawing on observations from fieldwork in two ethnic minority areas and a major literature review, we aim to address this gap and highlight the importance of ‘ethnicity’ as a relational category for any study of the deaf in China.

By taking into account ethnicity, this article diversifies research on deaf people in China, as well as speaking to increasing concerns within international Deaf Studies. Foss (2014, 428) has called for the discipline to consider in greater earnest ‘socio-economic class, region, religion, and other demographics of deaf people (see also Senghas and Monaghan 2002). Heeding this call we demonstrate how state-led ethnic classification impacts on deaf people’s lived socio-economic, linguistic and educational realities.

**Figure 1.**
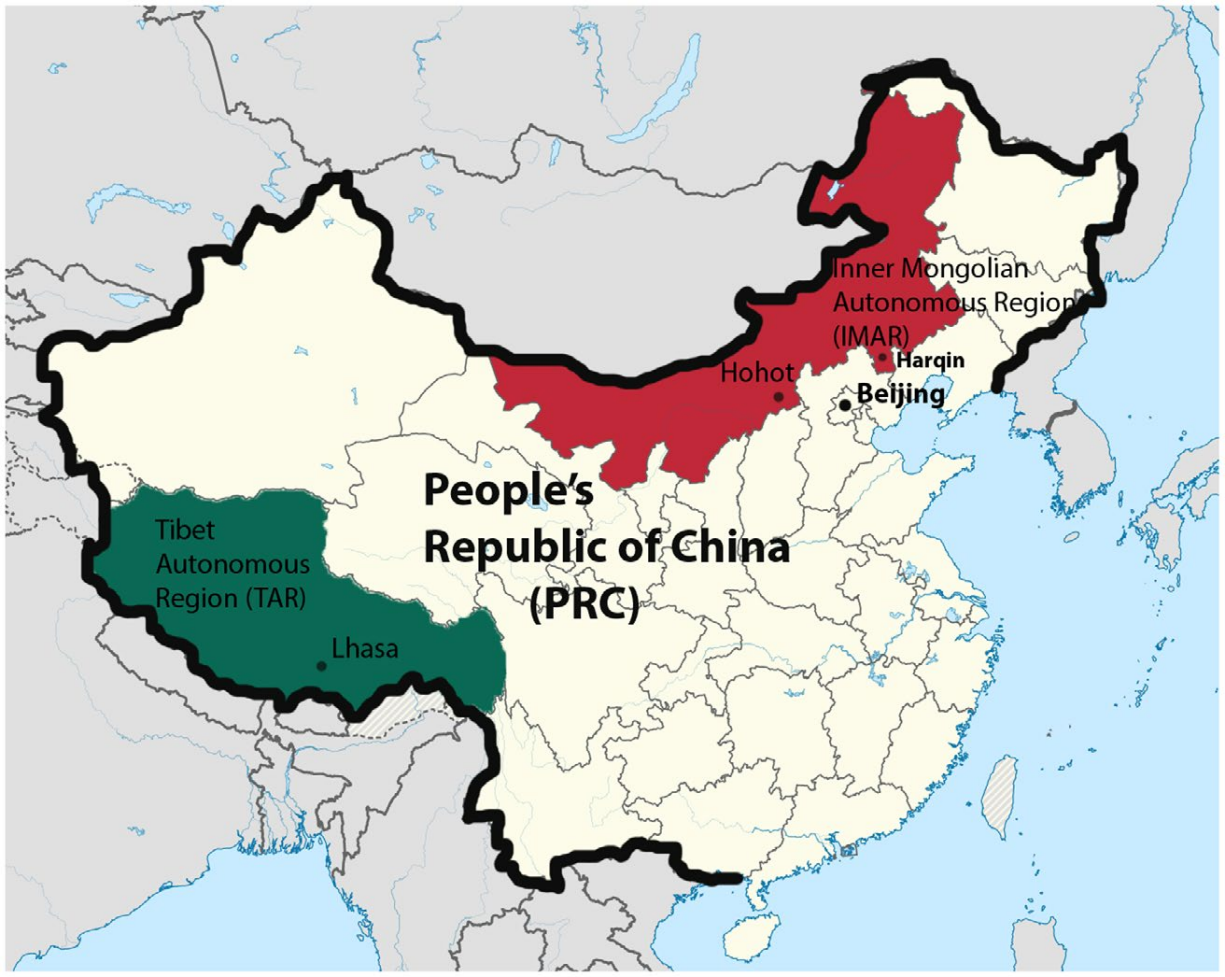
Map of the TAR and the IMAR within the PRC. Source: Wikipedia Map, adapted by Theresia Hofer.

We will focus on the lives of deaf Tibetans and Mongolians and policies relating to them in the Tibet Autonomous Region (TAR) and the Inner Mongolian Autonomous Region (IMAR). These two large provinces hold nominally autonomous political status within the PRC (Figure 1). The TAR (Tibet) is one of five provinces of the PRC with substantial Tibetan populations (altogether estimated to be about 7.5 million), while most Mongolians in the country (estimated to be about 5.8 million in total) live in the IMAR (Inner Mongolia).^3^

Comprising three main sections, this article examines issues important for deaf people within three major socio-political domains:

• Section I discusses policies and practices related to sign language use among the deaf in Tibet and Inner Mongolia.
• Section II focuses on educational policies and experiences of deaf children in local government Special Schools.
• Section III examines state welfare policies towards persons classified by the state as having ‘*tingli canji*’, or ‘hearing disability’.

We analyse observations in these three domains through what the anthropologist Stevan Harrell (1995) has called the state ‘civilising project’ of ethnic minorities[Bibr CIT0024]. Through this framework we enrich the understanding of deaf Tibetans and Mongolians within the Tibetan and Mongolian minority.

## The PRC state’s civilising project: historical background and political, linguistic and cultural dimensions

The PRC was founded in 1949. Crucial to understanding the PRC and the fate of the Tibetans and Mongolians within it is the PRC’s self-definition as a ‘unified multinational country’ (*tongyi de duo minzu guojia*). ‘A unified nation’ refers specifically to the PRC consisting of 56 *minzu* (‘nationalities’ or ‘peoples’). This number had been determined during the vast *minzu* classification project of the 1950s (Bulag 2002, 2010; Mullaney 2011). The designated 56 *minzu* include the Han Chinese,^4^ who form a clear majority and make up roughly 90% of the overall population of the country. The other 55 minority nationalities meanwhile account for the remaining 10% (see Mullaney 2011). Tibetans and Mongolians rank among the 10 largest minority *minzu*.

The creation of the PRC as a ‘unified multinational state’ has provoked a multitude of tensions, dilemmas and inequalities, especially among the country’s minorities. In 1995 Harrell published the edited volume *Cultural Encounters on China’s Ethnic Frontiers*. In its introduction, ‘Civilising Projects and their Reactions to them’, he theorises ethnicity as a means of codifying the relations of power between dominant groups at the centre – largely the Han Chinese – and subjugated populations, those classified as minorities in terms of a civilising project (Harrell 1995, 3–36).

The processes in which various *minzu* were defined, Harrell argues, were not merely descriptive or definitive (following criteria such as common territory, language, economy and common psychological make-up), they were also normative. Each *minzu* was placed on a scale of development, progressing from primitive, through slave, feudal and capitalist social organisation, to the ideal of communism with equitable distribution of the modes of production (Harrell 1995, 25). The ultimate goal was not only a successfully completed nation-building process but also a Communist ‘unified multinational country’. In this process, according to Harrell, the minority *minzu* were expected to be ‘improved’ and to meet standards set by Han Chinese (1995, 25). The Chinese party-state saw itself as a ‘Han’ institution, and as a helper to national minorities in their transformation. Preferential treatment policies for minority populations were enacted from time to time, such as their exemption from the nationwide one-child policy, tax benefits as well as preferential admission of minority *minzu* students to higher education.

Harrell follows a long tradition in anthropology of studying the relationship of nation-building, nationalism and ethnicity, and the ways in which these differ from nation-building in Europe and the United States during the nineteenth and twentieth centuries (for example, Eriksen 1993; Gellner 1983). China’s contemporary civilising project holds that all people are in theory equal as long as they work together towards Communism. In practice, however, the majority Han people are perceived as superior (Harrell 1995, 26). Intrinsic to the Communist civilising project, therefore, is that the minorities are perceived by Han Chinese to have languages and cultures that are in some ways backward and in need of development and transformation.

We find Harrell’s framework of the Han Chinese civilising project relevant for understanding how deaf Mongolians and Tibetans engage with state-led governing efforts. These range through resistance, collaboration and empowerment to assimilation of Han Chinese ways, a process that is conventionally known as sinification (Harrell 2001).

## Research issues: policies on language, education and disability in China

China is a linguistically diverse country and this has posed a considerable challenge to its nation-building, an issue we prominently discuss with regard to minority languages and minority sign language use in Section I. Even without the hundreds of languages spoken among the smaller ethnic groups, those defined as Han Chinese themselves speak an array of markedly different languages and dialects (Ramsey 1987; Sun 2006). Following establishment of the PRC, its Communist leadership considered it crucial to impose one national language, not only on the Han Chinese populations but throughout the entire country. Since the 1950s, the government has promoted a particular form of Chinese (based on Beijing Chinese), which is referred to as Putonghua (meaning ‘common language’).^5^ Putonghua has subsequently become a requirement for all official written and oral communication. Selected smaller languages have, however, also been granted official status of one kind or another and are allowed to be used alongside Putonghua in a number of ethnic ‘autonomous’ regions (Ramsey 1987). Tibetan language, for instance, is considered the second official language of the TAR and Mongolian that of the IMAR.

Similarly, the state has perceived the linguistic situation of the deaf in China and their diverse language practices as requiring unification and standardisation. In the 1950s the Chinese government devised so-called ‘experts on deaf education’ to establish a national sign language. In the process, language choices were made that closely related the new sign language to the syntax and the characters of Putonghua (Lin, de Garcia, and Chen-Pichler 2009), the result being Chinese Sign Language (CSL). Scholars debate the effects of the state promotion of CSL and it remains unclear to what extent CSL has actually superseded the sign languages or sign dialects of Shanghai, Beijing and other regions (Callaway 2000; Huang and Gu 2014; Xiao, Chen, and Palmer 2015; Xiao and Li 2013; Yang and Fischer 2002).

Regarding sign languages used by members of the 55 non-Han *minzu*, very little is known currently. Because language is a primary aspect of both ethnic classifications by the state as well as people’s own senses of belonging and relating to various ‘others’, sign language use in Tibet and Inner Mongolia will be an important thread in this article. What is the status and role of Tibetan and Mongolian sign languages? What new light does the current situation of sign language in these two ethnic minority regions shed on our understanding of the civilising project?

The educational sector, which is discussed in Section II, is a domain where satisfactory communication is a basic requirement. The existing literature on deaf education shows that throughout the history of deaf education in China, deaf schools in general tended to downplay the role of sign and show preference for other modes of communication (Callaway 2000, 68–72; Johnson 2003; Lytle et al. [2005] 2006; Yang 2008; Yang and Wang 1994). Notably, the situation for non-Han deaf children is not at all mentioned in this literature. Additionally, we need to consider that children in ‘autonomous’ regions have the stated legal right to gain an education in, or at least of, their native languages (Leibold and Chen 2014; Postiglione 1999; Tsung 2009). Again, the education of deaf children has not been discussed in the literature on educational rights and practices of non-Han children. To address this gap we discuss the use of language in deaf education. Is the promise to receive education in their own language also realised for deaf children in ethnic minority areas? Based on the education offered to deaf children in Tibet and Inner Mongolia, what conclusions can we draw for the nature and effects of the state civilising project?

In Section III we focus on the nature of national policies aimed at the so-called *canji ren* (‘disabled people’). This category and its related polices came into being in the late 1980s and are both closely tied to the work of the CDPF (Kohrman 2005). The CDPF, a large state-funded and state-run organisation, has adopted and consistently promoted a medicalised definition of disability (*canji*) in China, with rehabilitation and prevention as major approaches to disability. Owing to influence of the CDPF, most current disability policies are dominated by a medical approach, rather than the social model of disability championed internationally by various institutions and organisations working for the rights of people with disabilities (Oliver 1990). While former studies consider education and language to be central elements of the civilising project, disability practices and policies have previously not been taken into account. What role does the CDPF play in relating the medical model to deaf people in Tibet and Inner Mongolia? What are the implications of CDPF initiatives with regard to the civilising project in the two minority areas?

## Research methods: people and places

This article emerged through conversation and debate between the two authors, following their completion of fieldwork for earlier projects. Hofer draws on over a decade of anthropological field research experience in the TAR (see Figure 1) begun in 2003, where more recently she has carried out work with deaf Tibetans in the capital Lhasa, focusing on their emerging Tibetan Sign Language (TibSL),^6^ the government Special Schools and the lives of deaf Tibetans. In Lhasa it can be surmised from government data and Non Governmental Organisation (NGO) records that in 2006 between 1.65 and 2% of the TAR population were deaf or hard-of-hearing.^7^ Taking these figures together with the results of the 2000 population census yields an estimate of approximately 3100–3700 deaf and hard-of-hearing Tibetans living in Lhasa. Considering the relative absence of any sizeable urban centres in Tibet prior to the 1990s, this means that there is now a much greater number of deaf people living in close proximity to each other than ever before. This critical mass of signers, among other reasons, has contributed to the emergence and formalisation of TibSL, starting in 2000 (Hofer 2017). Apart from participant observation, Hofer’s research also included recorded audio-visual, qualitative interviews, analysis of government documents, NGO reports and various language materials, as well as artistic productions of her interlocutors.

Sagli meanwhile draws on research in Inner Mongolia, carried out in the context of a research project on poverty and disability.^8^ Her fieldwork took place in southeast Inner Mongolia, primarily in Harqin District, a largely rural area located about 80 km east of Hohhot, the capital city of Inner Mongolia, and 400–500 km northeast of Beijing (see Figure 1). Together with Chinese researchers, she made household visits and undertook interviews with people with disabilities, including some with so-called ‘speech and hearing disability’, as well as with members of their households.^9^ In addition, village clinics, local hospitals, a county rehabilitation centre, centres for the elderly and a Special School with deaf students were visited. This research also included a review of local and national documents on disability policy. According to the documents from local authorities in Harqin, the total ‘disability rate’ at the time of research was 6.3% (or 21,634 people), which is the same as the rate for China overall. Official statistics state that 3788 people in Harqin District (i.e. 1.1%) have ‘speech and hearing disability’.

Among the interviewees, some were Mongolian and some were Han. In Harqin District around 50% of the total population (342,000) consisted of people belonging to the Mongolian ethnic group, which is a high percentage for Inner Mongolia. Perhaps counter to the expectations of many readers, the population of Inner Mongolia today consists of 80% Han and only 18% Mongolians, the remainder made up by members of other *minzu*.

Drawing on this exposure and the empirical data gathered, our conversations and wider reading on ethnic minority issues within the PRC, we offer here a unique discussion of the policies and situations of deaf people in Tibet and Inner Mongolia. We were struck by certain differences as well as similarities between what we had observed in the two minority regions. Analysing and comparing these and then relating them to current debates in modern Chinese history and anthropology, we became acutely aware of a substantial gap in the wider literature. This related to understanding issues that cut across the socio-political categories of ‘ethnicity’ and ‘disability’, where we found that the effects and workings of the Han-Chinese civilising project had not been scrutinised with respect to sign languages, special education and disability policies.

## Section I. Local sign languages and ethnic minority policies

As a first step towards a more comprehensive understanding of the civilising project, we will focus on sign languages and ethnic minority policies. Particularly, we will examine the status of local sign languages in Tibet and Inner Mongolia, and explore how the language policies advocated by the state intersect with the use of local signing practices and local oral and written language among our research participants**.**

### Tibetan Sign Language in the making

In May 2004, a press release from China’s official news agency, Xinhua, drew attention to the emergence of TibSL. Titled ‘First Sign Language System Developed for Ethnic Deaf-mutes’ (Xinhua 2004), it reported that ‘Tibetan dactylology’ had over 700 popular signs, was developed by four members of the local deaf club and ‘differed from the one being used nationwide as the latter is basically a kind of Chinese characters conversion, whereas many Tibetans can neither read nor write the Chinese characters’ (Xinhua 2004). This official mention came on the back of several years’ work by local deaf Tibetans, including tangible evidence of the language they had produced such as sign language dictionaries, DVDs and children’s books.

**Figure 2.**
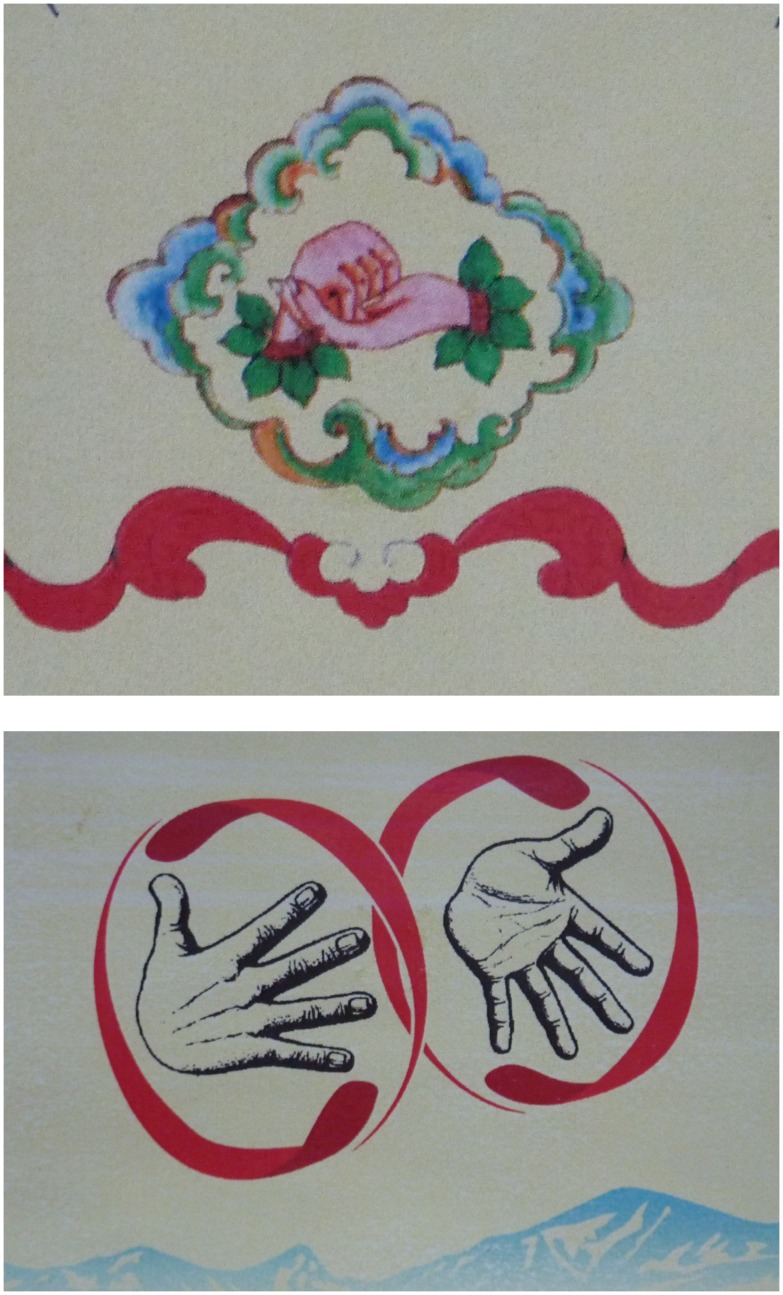
TIBETAN SIGN combining the sign for TIBET (and TIBETAN), which is a gesture for the eating of *tsampa*, with a sign for SIGN. © Photo by Theresia Hofer.

Beginning in 2000 and inspired by the international NGO Handicap International, a small group of deaf Tibetans had begun to document the lexical sign variation that existed among deaf Tibetans from different regions and backgrounds. Their aim was not, however, simply documentation. They intended also to reduce sign variations and produce some new grammatical innovations to allow for a more widely shared TibSL, a sort of *lingua franca* for deaf Tibetans (Hofer 2017; TDA 2011, 7). The TibSL project created several visual dictionaries, DVD volumes and also a Tibetan finger alphabet showing the shape of the Tibetan letters (TDPF and HI 2002; TDA n.d., 2005, 2011). Their publications use the English term ‘Tibetan Sign Language’, as well as the Tibetan term *bokyi lagda* and the Chinese term *zangyu shouyu*. Deaf signers use TIBETAN SIGN, combining the sign for TIBET (and TIBETAN), which is a gesture for the eating of *tsampa* (roasted barley flour, the staple food of Tibetans), with the sign for SIGN that is similar to International Sign for SIGN (Figure 2).^10^

Xinhua’s (2004) statement inadvertently acknowledged the usefulness of a local, Tibetan sign language for deaf Tibetans, because it suggested that learning and using CSL is difficult for deaf Tibetans, who often do not know how to read or write the Chinese characters on which much of it is based. Although not mentioned in the report, there is also a connection between TibSL, its fingerspelling and the written Tibetan language, as we discuss later in the educational context.

It is noteworthy that since 2008 several official policies have been passed at the level of the regional TAR government that explicitly recognise and support TibSL: one in Special Education in the TAR, and another addressing sign interpreting in courts and on television as well as in other public domains (CCP Tibet Autonomous Region Party Committee 2010). Yet despite such promising official statements and endorsements, in practice there has been scant use of TibSL in Special Schools or on television in the TAR, and neither have there been any state provisions for sign interpretation training. Since 2010, even simple TibSL classes for parents of deaf children and others have been scarce, and the abandoning of TibSL instruction as an extracurricular activity in the Lhasa Special School in the same year means that deaf children there are no longer exposed to TibSL.

In short, as a minority sign language, TibSL appears to be a language with some degree of official recognition as implied in the aforementioned policy documents as well as official and government-supported language materials, yet one also characterised by simultaneous emergence and endangerment (Hofer 2017).

### Speaking Chinese as the ‘ideal’ among deaf Mongolians in China

The situation of local signs and local sign language in Inner Mongolia that Sagli encountered was even more precarious than that in Tibet. All of the deaf and hard-of-hearing Mongolians she met in Harqin and Chifeng used Chinese and there was no indication of a local sign language in use or in the process of documentation. This is despite there being a Mongolian Sign Language in the neighbouring country, Outer Mongolia, and even though some deaf Mongolians in Inner Mongolia use local signs to communicate with one another. Therefore the lack of a more formalised Mongolian Sign Language among the research participants in Harqin and Chifeng does not necessarily indicate an absence of different forms of local, natural signs and gestures in communication between and with deaf people in Inner Mongolia. Rather, we have so far not found any evidence or language materials documenting local signs in Inner Mongolia.

The lack of a more developed or developing local sign language in Harqin reflects the wider situation of spoken Mongolian among the hearing population. In Harqin, Chinese is the most common written and spoken language. Only certain street signs, titles of public documents and other publications feature the Mongolian script, usually when carrying symbolic significance. During Sagli’s fieldwork, her interlocutors commonly remarked on how Han and Mongolians were no longer markedly different, neither in their way of living or in their language use.

The sinification and assimilation of the Mongolian population in Harqin is far from unique to that field site but typical for most places in Inner Mongolia, including rural areas (Bulag 2002). Over a period of several hundred years, Han Chinese – mostly poor peasants – have migrated to Inner Mongolia, searching for new farmland. Over time they became the majority in Inner Mongolia. In heavily Mongolian-populated areas, spoken Mongolian has remained dominant (at least until very recently), while in other areas the inhabitants – especially the young – have lost the Mongolian language (Burjgin and Bilik 2003). At least half of the officially counted ethnic Mongolians in China no longer know or speak Mongolian (Janhunen 2012, 11). Even though many inter-ethnic Chinese-Mongolian couples choose Mongolian ethnicity for their children, often with the hope of obtaining preferential treatment, Chinese is used in their homes and increasingly as a teaching medium in all schools. Given the surrounding linguistic assimilation of Mongolians in Inner Mongolia to Chinese, it is perhaps hardly surprising that there has not been a push, similar to the one in Tibet, to document and promote an ‘ethnic Mongolian sign language’.

### ‘Ethnic’ sign languages and the civilising project in Tibet and Inner Mongolia

The state civilising project shows itself as a concerted government effort to establish and promote written and spoken Chinese in conjunction with and through CSL, which similarly to Putonghua is posited as a ‘national language’ for all of the deaf in China. Through a national sign language, the deaf are thought to be best educated and thus integrated into the Communist project of national unification.

With regard to language use among deaf Tibetans and deaf Mongolians, we see that the sinification process has come far in Tibet but even further in Inner Mongolia. The impact of Chinese language appears stronger still for the deaf compared with the non-deaf living in these areas. Whereas spoken and written Tibetan and Mongolian share the status of official languages, there is no officially authorised Mongolian Sign Language in Inner Mongolia and TibSL has only very recently been treated on paper as such, yet its actual use and promotion remain very limited.

With regard to the situation in Inner Mongolia, we read the pervasive use of CSL, even among the deaf of an ethnic minority group with its well-established language and script, as an extension of the civilising project within China’s nation-building process. The lack of a more developed, documented and defended Inner Mongolian sign language to date is a reflection of the wider lack of self-consciousness among many Mongolians around the Mongolian language, culture and identity. An increasing influence of Chinese language and institutions is taking place despite Mongolian intellectuals’ attempts to maintain their language and culture (Bilik 1998, 2014; Burjgin and Bilik 2003).

The use and status of Tibetan and Mongolian sign languages reflect to some extent the respective situations of spoken Tibetan and Mongolian. Although spoken Tibetan has, since the early 1990s, been decreasing as a medium of instruction in Tibetan schools and in government domains, Tibetan is used in most Tibetan homes. This is no longer the case in Inner Mongolia.

Yet the emergence of TibSL over the past 15 years asks for a more nuanced reading than one of outright submission of deaf Tibetans to a process of sinification and assimilation. Rather than orchestrated by the government, TibSL’s formation bears the traces of the international disability movement. Handicap International worked to empower people with disabilities, and especially deaf people through sign language development, hence promoting a model of deafness that rests on ethnolinguistic difference rather than deafness as an impairment. Moreover, the Tibet Disabled Person’s Federation, the local counterpart to Handicap International, was a willing collaborator and supporter of TibSL-related projects and until early 2016 was headed by Tibetan cadres sympathetic to the cause of TibSL. The TDA and deaf Tibetans can therefore be argued to have, via the Tibet Disabled Person’s Federation, collaborated relatively well with PRC policy intentions, by seeking to incorporate the government’s *minzu*-related rhetoric of ‘one ethnic group – one language’ in ways that nevertheless gained TibSL a certain degree of official recognition alongside its increased legibility by the state.

## Section II. Sign language in education of the deaf

Given that proficiency in the language used in teaching is a key to successful education, one wonders in what languages deaf children access formal education. Hence, we will explore language use at Special Schools and in teaching of deaf children in Tibetan and Mongolian ethnic minority areas. How does their situation compare with the use of local minority languages in regular school education for hearing children?

### Learning Tibetan sign and learning to read and write Tibetan

**Figure 3.**
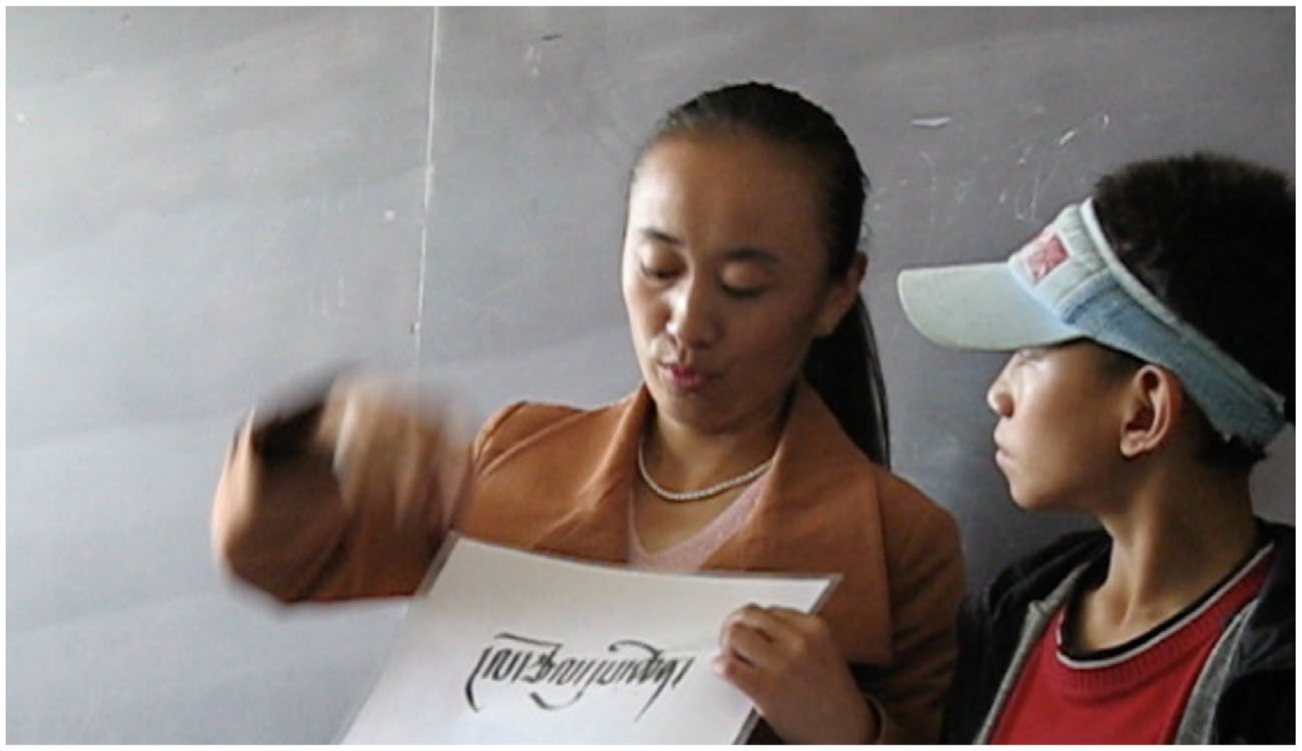
Teaching literacy in Tibetan via TibSL at the Lhasa Special School, 2007. © Photo by Theresia Hofer.

For most of its history the Lhasa Special School has been an almost exclusive Chinese language environment, with spoken Chinese and CSL as the main teaching mediums, and a clear focus on the acquisition of written Chinese. However, between 2000 and 2010 TibSL was also present in the classrooms, when volunteers from the TibSL project (with support from Handicap International) came to the government school each Saturday to teach TibSL and its fingerspelling as an extracurricular activity (Figure 3). The aim was to expose students to TibSL and to improve their literacy in written Tibetan through the use of TibSL fingerspelling for Tibetan letters, syllables and words (Hofer 2016).

During Hofer’s first visit, in one class comprising six to eight year olds the teacher was expanding their TibSL repertoire, training them in TibSL fingerspelling and (related to that) Tibetan reading skills. The slightly older class, of mainly eight to 10 year olds, was also taken up with practicing TibSL and TibSL fingerspelling. The aim of their exercises was to become able to fingerspell their Tibetan names in TibSL.

As with the younger class, these activities were meant to teach them new TibSL signs as well as to foster their proficiency in the Tibetan script. The culmination of their efforts at the end of the session was for each child to come to the front of the class and sign ‘I AM DEAF, MY SIGN NAME IS … IN TIBETAN SIGN WRITING, MY NAME IS …’ and then fingerspelling their Tibetan names in swift and delicate hand movements. Each student in the class was able, more or less, to complete this exercise successfully and was rewarded by everybody’s applause in sign – the holding up and shaking of their hands in the air.

**Figure 4.**
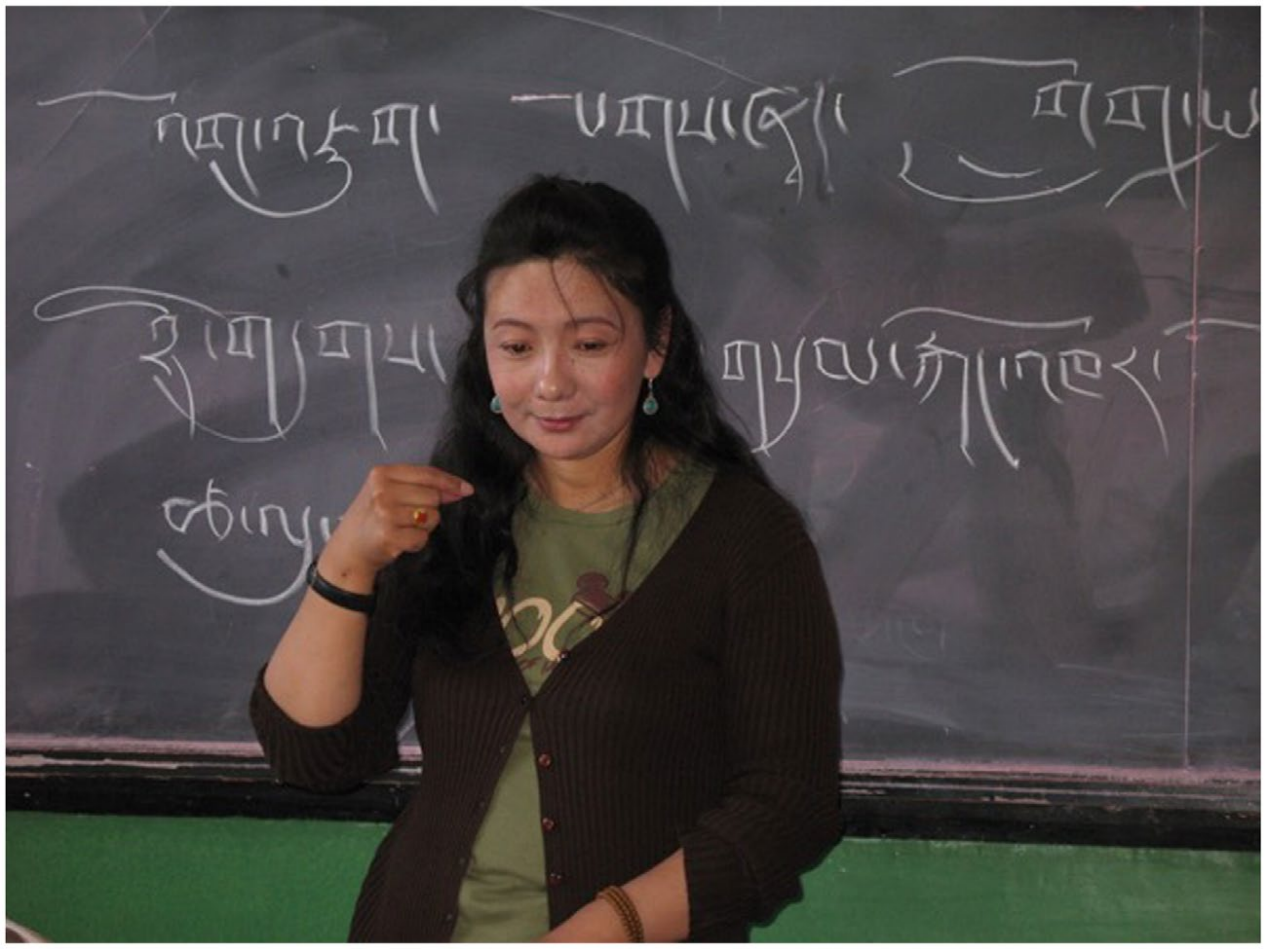
Teaching literacy in Tibetan via TibSL at the Lhasa Special School, 2007. © Photo by Theresia Hofer.

The oldest class that same morning was occupied with reading and writing Tibetan script, as well as practising the signs for the words they were learning to read and write. The teacher kept writing Tibetan vocabulary – shirt, fur hat, umbrella, tricycle, orange – on the blackboard and the pupils copied them into their notebooks, having repeated the TibSL signs after their teacher (Figure 4). Clearly, in all three classes, the TibSL fingerspelling of Tibetan letters, syllables and words served as a bridge to learn reading and writing Tibetan and become at least a little familiar with its arcane and complex orthography.

At the time, a TDA and China Deaf Association collaborative effort was also underway. This resulted, in 2010, in a document outlining a comprehensive and combined TibSL/CSL curriculum aimed at improving deaf Tibetan teenagers’ and adults’ literacy in both Tibetan and Chinese (TDA 2010). Although not yet in use, this curriculum, if itself slightly adapted and the government teachers duly instructed in TibSL, could offer a unique opportunity to the students of the Special Schools in the TAR to effectively gain basic literacy in Tibetan.

### Special School Education for the Deaf in Harqin

In contrast to the Lhasa Special School, students at Chifeng Special School in Inner Mongolia have never benefitted from exposure to a local sign language – neither as a medium of instruction nor as a means to learning spoken or written Mongolian. In two of the Harqin households where Sagli and her Chinese research colleagues undertook interviews, there were young boys who, according to official definitions, had ‘hearing and speech *canji*’. Jin, aged five, and Ming, aged six, although not yet of regular school age (six years), had been receiving a pre-school education for almost two years.^11^ Both boys were in a relatively privileged position, because their families had received financial support from the local disability organisation for the school fees.

Jin and Ming were boarders at Chifeng Special School. At this school the teaching medium in all classes was spoken and written Chinese, sometimes assisted by the use of CSL signs. This was despite the school being categorised as a *minzu* (ethnic minority) school and the boys belonging to the Mongolian *minzu*. However, for Jin and Ming it was nothing exceptional that spoken Chinese, rather than any local spoken or signed language, was the standard teaching medium. Chinese was also the common language used within the two boys’ families. To improve Ming’s reading skills, his parents had decorated the walls surrounding his bed at home, not with Mongolian script or pictures of signs, but with Chinese characters.

That spoken Chinese was the teaching medium in a Special School in a minority area was also not at all exceptional. According to Sagli’s research partners, none of the 45 Special Schools in Inner Mongolia uses either Mongolian or Mongolian sign as a teaching medium or even as a subject. This shows that for deaf children attending local Special Schools, the right to be educated in, or at least learn, the language of their own ethnic group is not currently fulfilled.

Jin and Ming’s parents explained that the boys received a combination of training in speech, lip-reading, fingerspelling and signing at the Chifeng Special School. The school also offered short signing courses to parents, but they tended to prioritise the boys’ speech training. Both sets of parents hoped that one day the boys would be able to go on to a regular school. Their aspirations were thus in line with the recent official policy of ‘learning in regular classes’ (*suiban jiudu*); that is, the inclusion of deaf pupils – and indeed children with any disability – in regular primary and secondary schools (Deng and Guo 2007; Malinen 2013; Xu 2012).

### Educating as civilising in Tibet and Inner Mongolia

The education of non-Han people has been shown to be an important component of the civilising project (Hansen 1999a, 1999b). Even if minority languages are still taught and spoken within many government schools in ethnic minority areas, the whole education system in Tibet and Mongolia is evidently changing towards extensive use of Chinese as a teaching medium (Bilik 1998; Ma 2014; Postiglione 1999; Tsung 2009). Deaf students in Tibet and Inner Mongolia are effectively already receiving their entire education in spoken and written Chinese, and not in Tibetan or Mongolian. We found that even though the nationwide special education curriculum was adapted at the Lhasa Special School, where they offer two classes teaching Tibetan written language a day, most of the deaf students attending these classes fail to become literate even in basic Tibetan. In the past, only the voluntary, extracurricular teaching of TibSL at the Lhasa Special School set a precedent for deaf Tibetans succeeding in becoming literate, at least to some extent, in written Tibetan. Nowadays, if sign language is used in teaching at Special Schools in Tibet and Mongolia it is only CSL.

Since the 1990s, national policies have been encouraging children with disabilities to ‘learn in regular classes’, which represents the latest turn in educational policy for people with disabilities in China (McCabe 2003; Xu 2012; Yang and Wang 1994). In most cases, this implies that deaf students will have to lip-read and use Chinese to speak and write. Sign language interpreters are rarely available.

The situation for deaf students to learn or be educated in their own ethnic language (either in local sign or written Tibetan or Mongolian in school) is therefore worse than the already problematic situation of regular local language education in ethnic minority areas. In effect, whether by design in the Special Schools or by default in regular schools, deaf students in Tibet and Inner Mongolia are more likely to gain a lesser and lower quality education. They therefore continue to have greater difficulties in communication, and as adults often experience the long-term consequences of a compromised schooling, such as unemployment, low-paid jobs and exploitation by employers (Li and Zhao 2008).

## Section III. Disability policies and rehabilitating the Deaf

Disability policies are yet another political domain that has impact on the lives of many deaf. Compared with sign language and educational policies, this area has so far received far more political attention as well as financial resources. In the only major ethnography of disability in China to date, Kohrman (2005) has demonstrated how the establishment and rise of the CDPF since the late 1980s has gone hand-in-hand with a highly politicised process, in which various forms of *canji*, or ‘disabilities’, were categorised according to medical criteria. The deaf were redefined as persons with ‘hearing and speech disability’ (*tingli yuyan canji*), and as such became one of the five categories of *canji* featured in the first nationwide survey of disabilities in 1986 [ (2005). The Chinese government had in fact helped found a China-wide organisation to assist the blind and the deaf during the 1950s, but this association was subsequently subsumed under the CDPF [ (2005), whose stated mission is as an organisation for all people with disabilities. Today the CDPF is commissioned by the party-state to develop and implement national disability policies and to carry out national surveys of disability.^12^

Although the CDPF has also played a key role in initiating legal protection of people with disabilities in China (CDPF 2014; Kohrman 2005; Stein 2010), its approach to disability has been highly medicalised. The CDPF has championed modern medical rehabilitation services for people in China, including for the deaf. Since the 1990s’ ‘Three Rehabilitations Project’, the CDPF has promoted ‘oral-language training for deaf children’ as a rehabilitation intervention alongside non-deaf specific ‘cataract surgery’ and ‘polio-correction surgery’ for people with other disabilities (Kohrman [, 2005, 127); likewise, ‘hearing and speech’ training is categorised as ‘rehabilitation’ (CDPF 2014). This medical approach has continued to expand as new technologies become available, and nowadays encompasses the distribution of advanced hearing aids, cochlear implants and other technological innovations (CDPF 2014).

Rehabilitation is only one of the main approaches and priorities of the Chinese state towards people with disabilities, another being the prevention of disabilities (Fjeld and Sagli 2011; Stone 1996). This approach was already formulated in 1979 when a new population policy – known internationally as the ‘one-child policy’ – started to be implemented (Greenhalgh and Winckler 2005; Stone 1996; White 2006). Less well known is that this policy had two main aims: its purpose was not only to reduce the quantity of the population, but also to increase its quality through eugenic measures. It implied that ‘impairments’ were considered unwanted and that disabilities should be prevented if at all possible (Dikötter 1998; Fu et al. 2010). The prevention of impairments has been powerfully enforced and many resources supplied for the development and later massive distribution of advanced prenatal testing technologies (White 2006). Today, such technologies are extensively used all over China, even in remote rural regions. Related to this, a significant quantity of research aimed at detecting ‘deafness genes’ has been carried out including, in minority areas.

### Medicalising deafness in Tibet and Mongolia

Our experiences from the field sites in Tibet and Inner Mongolia reflect the state’s strong medical orientation towards deafness. The rehabilitation paradigm is still dominating pre-school training for deaf children, where such facilities exist. According to the CDPF there are rehabilitation centres for deaf children and grassroots hearing and speech training institutions nationwide (CDPF 2014).

Not only doctors and researchers promote the medical model of disability in China. Also among families of deaf children the view that deafness may be remedied through medical means is widespread (Callaway 2000). We encountered this attitude often during fieldwork in both Tibet and Inner Mongolia. The irony of this situation is that a high proportion of the deaf, at least in Tibet, have become deaf due to the (mis)use of medicine (mainly aminoglycoside antibiotics) in the first place.^13^ Such antibiotics – widely available from government and private facilities and used liberally – are available even without prescription, and where prescribed are often given for the wrong indications and/or at the wrong dose. This problem is especially urgent in rural areas with poor health care infrastructure undergoing neoliberal health reform (Hofer 2011). The interviews in Inner Mongolia and Tibet also revealed that all of the families with deaf or hard-of-hearing members said they had sought medical cures and diagnoses from a great number and variety of hospitals and doctors with biomedical, Chinese-medical and folk-medical orientations, travelling near and far to access them.

Despite the main cause of deafness being mainly medical, Tibetans and Mongolians have not been exempted from biomedical research projects aimed at identifying and studying ‘deafness genes’ with the aim to prevent genetically transmitted deafness. In Inner Mongolia, children at Chifeng Special School have been recruited for studies investigating genetic causes of hearing impairment (for example, Dai et al. 2008), and in Tibet, Lhasa Special School students have also been targeted for such research (for example, Yuan et al. 2012).

With the current emphasis on genetic causes and medical cures of deafness, the state, families and educators currently look away from the health care professionals’ responsibility for causing deafness as well as from improving communication once deafness has occurred. The TDA president has taken issue with this privileging of medical cures for deafness, instead asserting that better communication, sign interpretation and capacity-building for deaf people are key to improving their livelihoods (Unal 2010). Nonetheless, while this is a widely accepted view among deaf Tibetans, it would be a minority opinion among hearing Tibetans, medical researchers and civil servants.

Aimed at poverty reduction, the CDPF provides poor deaf children with free cochlear implants and free hearing aids; obviously, however, this programme does not reach every poor household. In Tibet we learned that hearing aids had been distributed to some deaf children (no cochlear implants have yet been given out there) but proved either ineffective or stopped working due to technical issues or simply for lack of appropriate batteries.

These programmes aimed at poor people with disabilities are particularly relevant for ethnic minority regions because many of these, especially the rural areas, are poverty stricken. Harqin, for example, is officially designated as a ‘poverty county’. Paradoxically, however, the medical approach to deafness is one of the factors that generate poverty. Families’ prioritising of medical solutions in many cases leads to their impoverishment due to the high cost of extensive medical treatments (Sagli et al. 2012).

### Medicalising and civilising

With regard to the civilising project, the most important consequence of the medical approach to deafness is that signing and the promotion of sign language become overshadowed by hearing and speech training, as well as by technical solutions. Furthermore, it is not a local spoken or written language that it used in such training, but rather it is the national language, Putonghua. The education of non-Han people in Chinese language therefore occurs not only in the nation’s Special Schools but also in rehabilitation centres. The medical and rehabilitation approach to deafness facilitates the civilising project, because it results in decreased use of local sign languages while at the same time increasing the use of Putonghua.

The spread of advanced hearing aids and other forms of bio-technology associated with the medical approach, including genetic research, is also clearly in line with the spirit of the civilising project. Science and technology are areas where the state perceives ethnic minority peoples to be particularly in need of progress. Being deaf and a member of an ethnic minority generates the possibility of being considered ‘doubly backwards’, and hence using technology (rather than acceptance and education) to treat deafness has become an effective means to advance ethnic minorities towards Han norms and standards.

To conclude, we need to point out that it is a modern, educated, ‘cultured’ Han that sets the standard in the civilising project and that needs to be emulated by members of ethnic minorities, not any way of being Han (Harrell 1995, 2001). Following from this, we suggest that the normative body needs to be added to the list of norms promoted by the civilising project. To be hearing and speaking (ideally Putonghua) complies with this norm and standard, while being hard-of-hearing or deaf and signing does not. These are even seen as promoting further ‘abnormalities’ such as a lack of education, for example. In China, the internationally championed Deaf-Pro conception of being Deaf is therefore rarely regarded by the PRC state as unique and worthy of celebration. In fact, such a positive conception of Deafness would go strongly against current political trends in the PRC.

## Conclusions

Focusing on the situation for deaf Tibetans in Tibet and deaf Mongolians in Inner Mongolia, this article has examined the three socio-political domains of language, education and disability policies. We have demonstrated that in public life, both in Tibet and Inner Mongolia, the role of local minority sign language is either very limited (Tibet) or completely absent (Inner Mongolia). TibSL is so far the only minority sign language in China with some degree of official recognition. Yet, while oral and written Tibetan and Mongolian have official status and are studied in government educational facilities by hearing children, TibSL is used only outside of the formal educational sphere. The legal right of minorities to be educated in their own minority language is therefore not realised for deaf children in Tibet and Inner Mongolia. This is either because of lack of local sign languages in formal education or because the children are not able to gain literacy in local written languages. In addition, disability policies also promote use of Putonghua as deaf children are taught to read and speak Chinese in rehabilitation centres. In sum, state institutions expect the deaf in Tibet and Inner Mongolia to learn written and to some extent spoken Putonghua.

To better understand the roots of the strong emphasis on learning Chinese, we have analysed the situation in Tibet and Inner Mongolia in the light of the ongoing Han civilising project of ethnic minorities. Language and education are important components in the civilising project (Hansen 1999a, 1999b; Harrell 1995, 2001). To understand further dimensions of the civilising project, we have offered an analysis of sign language use and special education for non-Han deaf students as well as disability and rehabilitation policies directed at deaf people at large. The peoples in the ethnic minority regions, whether hearing or deaf, have their lives and life opportunities shaped by the tension between powerful national policies aimed at unifying the nation and policies intended to preserve only carefully selected aspects of minority cultures, languages and other so-called ‘ethnic characteristics’. Within the civilising project the preservation of minority languages and cultures is not a goal in itself. It is perceived merely as a phase in development towards ultimate ‘civilisation’. Government incentives in support of more, official *minzu* sign languages are therefore unlikely to be introduced. On the contrary, it is in full compliance with the ‘civilising’ mission to expect the deaf to learn Chinese and CSL rather than minority (sign) languages.

In both Tibet and Inner Mongolia, the deaf suffer from a lack of opportunities to learn their own ethnic sign language or the written minority languages of the regions. Future research could shed valuable light on the consequences of this situation as important questions remain. For example, when a language is considered both by the state and ethnic minorities as a key component enabling its members to access local cultural knowledge and participate in the formation and maintenance of ethnic identity, does this deficiency have consequences for the senses of ethnic, and perhaps national, belonging among deaf Tibetans and Mongolians? What about the well-being of such groups of people?

As well as presenting findings and analyses of policies and conditions specific to the PRC and within it, Tibet and Inner Mongolia, this article also contributes to current debates in international Deaf Studies. Within that field, scholars are reminding us that the deaf, when perceived as a cultural group, vary across a range of identity-related and demographic categories (Foss 2014, 428). Ethnicity is one such factor that has largely been ignored in Deaf Studies. We see it as not only fruitful but also necessary to discuss the governance of linguistic, educational and bodily differences in Tibet and Inner Mongolia in the context of the governance of ethnic differences. Accordingly, and from this study in China, we see a need to consider not only ‘socioeconomic class, region, religion, and other demographics’ (2014, 428) but also the specific socio-political value and meaning of such variables, including ethnicity.

## Disclosure statement

No potential conflict of interest was reported by the authors.

## Funding

The research for this article was supported by Research Council of Norway [Grant 190096]; European Commission, Marie Słodowska-Curie Individual Research Fellowship [Grant 303139]; Wellcome Trust, Research Fellowship [Grant 104523].

## Notes

1. The authors are aware of the international convention of using ‘deaf’ to denote physiological deafness, ‘Deaf’ to highlight cultural identity and ‘d/Deaf’ for the often inherently mixed nature of audiological and socio-cultural conditions (Senghas and Monaghan 2002). For readability’s sake as well as the limited applicability of these kinds of distinctions in the Global South, they have been omitted and the term ‘deaf’ in lower case is used.

2. Again, for ease of reading, from now on we use ‘China’ interchangeably with the ‘People’s Republic of China’ or the ‘PRC’.

3. For simplicity’s sake we will refer to the TAR as ‘Tibet’ and the IMAR as ‘Inner Mongolia’, and will do so without any intended reference to the larger geographical and cultural entities that these terms can also indicate.

4. ‘Han’ itself is a constructed category, and a relatively recent one at that. See (Gladney 1991, 81–87) and Harrell (2001, 295–296).

5. It is also commonly referred to as Mandarin or Standard Chinese. For ease of reading, we use Chinese interchangeably with Putonghua throughout the article.

6. The abbreviation TibSL is used to distinguish the language from other sign languages that are already referred to with the abbreviation of TSL, such as Taiwanese Sign Language and Thai Sign Language.

7. The 2000 census states that the total population of Tibet was 2.6 million, with 94% of Tibetan ethnicity (TIN – Tibet Information Network 2003). Summarising the 2006 nationwide government survey of people with disabilities, Handicap International and the Tibet Deaf Association (TDA) state that there were about 45,000–50,000 deaf in Tibet (Handicap International 2011, 6–7). The total percentage of those with ‘hearing disability’ in Tibet was 1.64%. According to the survey, Tibet is home to 194,000 people with some form of disability, 26% of whom are classified as have a ‘hearing and speech disability’ (*tingli yuyan canji*). There are no Lhasa-specific figures available.

8. This project was titled ‘Poverty, Disability and Access to Social Welfare in the People’s Republic of China’ (2009–2012). See Sagli et al. (2012).

9. Members of 20 households in the Harqin area were interviewed, first in March 2010 and then a second time in June of the same year; eight of those had a member with a so-called ‘speech and hearing disability’.

10. Capital letters will be used for terms that render TibSL signs.

11. These names are pseudonyms.

12. The last survey was conducted in 2006/07, when the ‘hearing and speech disability’ (*tingli yuyan canji*) category was split into two: ‘hearing disability’ (*tingli canji*) and ’speech disability’ (*yuyan canji*; CDPF 2008). In this survey, *canji ren* were classified and numbered accordingly: hearing disability (20.04 million), visual disabilities (12.33 million people), speech disability (1.27 million), physical disability (24.12 million), intellectual disability (5.54 million), mental disability (6.14 million) and multiple disabilities (13.52 million) (CDPF 2008).

13. A survey, carried out in 2008, of the local Health Bureau in Lhasa and Shigatse Prefectures indicated that about 70% of the deaf people interviewed had become deaf due to having received incorrect medication – either in type and/or dose (T. Hofer, personal conversation with TDA and Tibet Disabled Person’s Federation, Lhasa, 2016).
